# Correlation among high salt intake, blood pressure variability, and target organ damage in patients with essential hypertension: Study protocol clinical trial (SPIRIT compliant)

**DOI:** 10.1097/MD.0000000000019548

**Published:** 2020-04-03

**Authors:** Wei Cai, MingJian Lang, XiaoBo Jiang, Qian Yu, Congliang Zhou, Wenshu Zou, Xiaohua Zhang, JianGuo Lei

**Affiliations:** a Department of Cardiology, The Affiliated Chengdu Fifth People's Hospital, North Sichuan Medical College; b Department of Cardiology, Chengdu Fifth People's Hospital, Chengdu, Sichuan Province, China.

**Keywords:** blood pressure variability, essential hypertension, high salt intake, target organ damage

## Abstract

**Background:**

Essential hypertension is a multifactorial disease, which is affected by genetic and environmental factors, and can cause diseases such as cerebrovascular disease, heart failure, coronary heart disease, and chronic renal failure. High salt intake is a risk factor for hypertension, stroke, and cardiovascular disease. Blood pressure variability (BPV) is a reliable independent predictor of cardiovascular events and death. At present, there are few studies about the correlation among high salt intake, BPV, and target organ damage (TOD) in patients with hypertension.

**Objective:**

The purpose of this study is to compare 24-hour urine sodium excretion, BPV, carotid intima–media thickness, left ventricular mass index, and serum creatinine or endogenous creatinine clearance rate. To clarify the relationship between high salt load and BPV and TOD in patients with hypertension.

This study is a cross-sectional study. It will recruit 600 patients with essential hypertension in the outpatient and inpatient department of cardiovascular medicine of Chengdu Fifth People's Hospital. Researchers will obtain blood and urine samples with the patient's informed consent. In addition, we will measure patient's blood pressure and target organ-related information.

**Trial registry:**

The study protocol was approved by the Chengdu Fifth People's Hospital. Written informed consent will be obtained from all the participants. The trial was registered in the Chinese Clinical trial registry, ChiCTR2000029243. This trial will provide for the correlation among high salt intake, BPV, and TOD in patients with essential hypertension.

## 1 Introduction

Essential hypertension is a common circulatory system disease, which is affected by both genetic and environmental factors,^[1]^ and accounts for more than 40% of the cardiovascular disease total burden.^[2]^ High salt intake can not only increase blood pressure, but also reduce the efficacy of antihypertensive drugs. And 24-hour urine sodium test is the best way to measure an individual's daily sodium intake.^[3]^ Blood pressure variability (BPV) is a basic characteristic of blood pressure and can reflect the magnitude of blood pressure fluctuations over a period of time. Recent research shows that BPV can better reflect cardiovascular activity than blood pressure levels and is more closely related to target organ damage (TOD) in hypertension.^[4–6]^ Failure to detect and treat essential hypertension early can damage important organs such as the heart, brain, and kidneys, causing diseases such as left ventricular hypertrophy, atherosclerosis, and renal failure.^[7,8]^ Left ventricular hypertrophy (LVH) is an independent cardiovascular risk factor in patients with essential hypertension.^[9,10]^ Intima–media thickness (IMT) is a marker that can be used to assess the severity of atherosclerosis.^[11]^ Serum creatinine, endogenous creatinine clearance rate (Ccr) and 24-hour urine microalbumin (MA) were commonly used indicators of renal function. However, the relationship among high salt load, BPV, and TOD in patients with hypertension is unclear. This study recruited patients with essential hypertension in the Department of Cardiovascular Medicine of Chengdu Fifth People's Hospital. Collect basic patient information, blood pressure measurements, blood specimens, 24-hour urine specimens, and other clinical examination results. Carotid IMT, left ventricular mass index (LVMI), serum creatinine or Ccr, 24-hour urine MA, and other indicators were used to evaluate TOD. To clarify the relationship among high salt intake, BPV, and TOD. To lay a good foundation for early detection of TOD in essential hypertension and related measures.

## 2 Materials and methods

### 2.1 Aim of the study

The objective of this study is to investigate the relationship among high salt intake, BPV, and TOD in patients with hypertension. Through laboratory inspection, echocardiography, and ambulatory blood pressure monitoring, we could identify risk factors and take steps to reduce the risk of damage to the patient's target organs.

### 2.2 Design and registration

**Figure 1:**
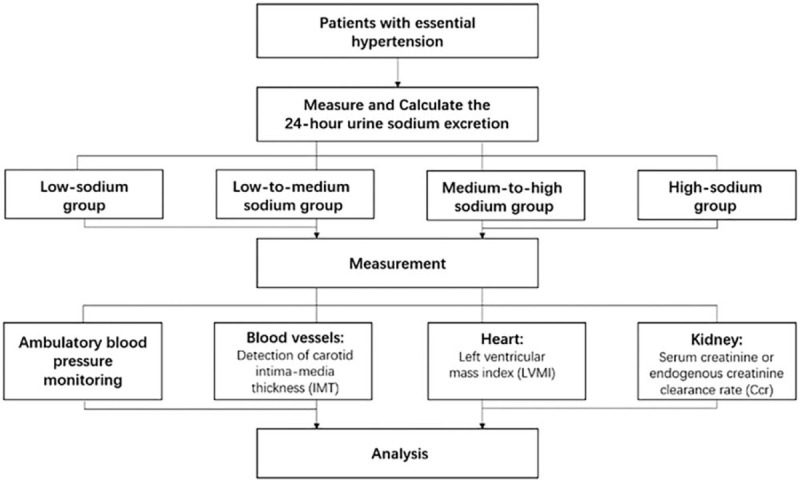
Flow chart of protocol. Ccr = creatinine clearance rate, IMT = intima–media thickness.

This trial was registered in the Chinese Clinical trial registry, ChiCTR2000029243. The flow chart of this study is shown in Figure 1. This cross-sectional study will be conducted at the Fifth People's Hospital of Chengdu City, Sichuan Province from April 2020 to March 2022.

### 2.3 Participants and eligibility

#### 2.3.1 Inclusion criteria

##### 2.3.1.1 Participation of the population with primary hypertension

Outpatients and inpatients with primary hypertension are over 18 and are not limited in gender. The diagnostic criteria for hypertension are based on 2010 Chinese guidelines for the management of hypertension.

##### 2.3.1.2 Exclusion criteria

Participants who will meet any of the following will be excluded:

(1) Acute or chronic renal insufficiency: eGFR ≤45 mL/minute
(2) Severe cardiovascular disease
(3) Exclude patients with malignant tumors, hematological diseases, rheumatic immune system diseases
(4) Heart failure (systolic)
(5) Take routine dose or overdose of diuretics continuously for 2 weeks (but the fixed compound of RASI + 12.5 mg thiazide diuretics can be included).

### 2.4 Recruitment

All patients consisted of outpatients and inpatients with essential hypertension in the Department of Cardiovascular Medicine of Chengdu Fifth People's Hospital from July 2017 to July 2018. Consent will be obtained by the investigators with the principle of informed consent, which confirms the participants’ voluntarism. The participants will sign the consent form that would include the information on the use of their blood sample, urine sample, ambulatory blood pressure data, and echocardiography results.

### 2.5 Outcome measures

According to the schedule depicted on Table 1, we will use a series of outcome measures.

**Table 1:**
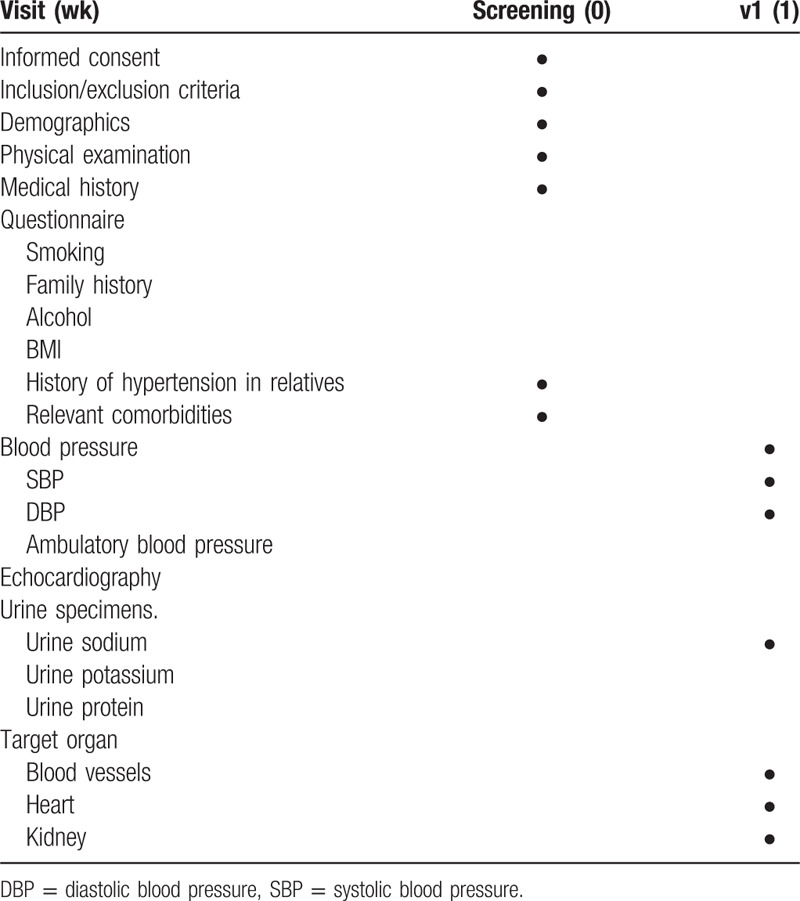
Study schedule for data collection and outcome measures.

#### 2.5.1 Questionnaire

The participants will complete a questionnaire that would include general sociodemographic information (e.g., age and sex), personal habits (e.g., smoking, alcohol consumption, sleep pattern, and dietary habits), anthropometric measurements (e.g., height, weight, and BMI), pregnancy, menstrual pattern and history, history of present illness, medication history, history of hypertension in relatives, and relevant comorbidities (e.g., cerebrovascular disorders, heart failure, coronary disease, chronic renal failure, and aortic disease).

#### 2.5.2 Blood pressure determination

The patient begins to measure blood pressure after resting for 5 minutes in a quiet room. No matter what position the patient is in, the upper arm should be placed at the heart level. The systolic blood pressure (SBP) and diastolic blood pressure (DBP) were determined using the Corson sounds 1st and 5th, respectively, and each person's blood pressure was measured at least 2 times at intervals of 1 to 2 minutes. If there is a big difference between the 2 measurements, we will measure again.

#### 2.5.3 Ambulatory blood pressure monitoring

All subjects used a fully automatic noninvasive cuff blood pressure monitor (fixed model) for blood pressure monitoring. The cuff is fixed to the subject's left upper arm and can be used for daily work and activities, but strenuous exercise should be avoided. Monitoring time: measured every 30 minutes during the day (8:00–22:00), every night at 22:01 to 7:59 the following day, every hour. The daily effective blood pressure reading is greater than 85% of the set number, and the effective value needs to be greater than 48 times. Effective blood pressure readings: SBP 70–260 mm Hg (1 mm Hg = 0.133 kPa), DBP 40 ∼ 150 mm Hg, pulse pressure 20 ∼ 120 mm Hg. Monitoring indicators: 24-hour mean SBP (24-h SBP), 24-hour mean DBP (24-h DBP), day-time mean SBP, night-time mean SBP, day-time average DBP, and night-time mean DBP. The blood pressure standard deviation (SD) and its coefficient of variation (CV) at each time period were used as indicators of BPV at that time period. (CV-SBP, coefficient of variation of systolic blood pressure) CV is the ratio of the SD to the mean. Expressed by the formula: CV = σ/μ. Monitoring normal reference value: 24 hour mean blood pressure <130/80 mm Hg, daytime blood pressure <135/85 mm Hg, nocturnal blood pressure <120/70 mm Hg. Blood pressure circadian rhythm = (average blood pressure during the day − average blood pressure during the night) / average blood pressure during the day; >10% is normal blood pressure circadian rhythm (dipper type hypertension), otherwise it is nondipper type hypertension, <0 is the anti-dipper type hypertension; CV-SBP is grouped with a cutoff of 12%.

#### 2.5.4 Collection and determination of 24 hour urine specimens

Keep a normal diet and avoid strenuous exercise before urine retention. Record the start and end time of urine retention and use a wide-caliber container to collect urine for 24 hours. After mixing, measure and record the total urine volume. The urine sodium, urine potassium, and urine protein were measured in the laboratory of Chengdu Fifth People's Hospital and the 24-hour urine sodium excretion and 24-hour urine MA volume were calculated.

#### 2.5.5 Target organ measurement standard

1. Blood vessels: Detection of carotid IMT: The patient lies on his back and turn the head to the opposite side. The distance between the intimal surface of the common carotid artery bifurcation 1 cm non-plaque and the outside of the median was measured, and the maximum value was taken on both sides. According to the relevant diagnostic criteria in the 2010 Chinese guidelines for the management of hypertension, the carotid intima media thickness >0.9 mm is defined as thickening and the local protrusions protrude from the arterial lumen >0.5 mm or IMT > 1.5 mm is defined as carotid atheromatous plaque.
2. Heart: LVMI: Measure the left ventricular end diastolic diameter (LVDd), interventricular septum, and left ventricular posterior wall, measure 3 consecutive cardiac cycles and take the average. According to the Devereux formula, calculate left ventricular mass (LVM) and LVMI: LVM (g) = 0.8 × 10.4 × ([IVST + PWT + LVDd]^3^ − LVDd^3^) + 0.6; LVMI = LVM / body surface area.
3. Kidney: Serum creatinine or endogenous Ccr: 24-hour urine A. MA is defined as negative urine routinely detected protein, and urinary albumin excretion rate is 30 to 300 mg/24 hour.

#### 2.5.6 Grouping criteria

Twenty-four-hour urinary sodium was used to estimate sodium salt intake. Based on the quartiles of the 24-hour urine sodium, the enrolled population was divided into low sodium group, low-to-medium sodium group, medium-to-high sodium group, and high sodium group.

### 2.6 Data collection, management, and quality control

To maintain the quality of this trial, and data management including data collection, validation, and completion will be conducted by the Fifth People's Hospital of Chengdu City, Sichuan Province, China. To ensure that outcome assessments are of a high standard in accordance with the trial protocol, the investigator and the assistants will attend a 6-hour training workshop before the initiation of the trial. The investigator and the assistants will also be provided with a written protocol and standard operating procedure documents. All the data will be checked regularly by clinical trial coordinators. The study subjects were selected in strict accordance with the inclusion and exclusion criteria. Blood pressure measurements in patients with hypertension are performed by trained cardiovascular physicians.

### 2.7 Statistical methods

We will use SPSS 17.0 software for statistical analysis in this study. Normal distribution measurement data was expressed as mean ± SD. One-way analysis of variance was used for comparison of means among multiple groups, and SNK test was used for pairwise comparison. The measurement data of non-normal distribution is expressed as median M (P25–P75). After the natural logarithm is converted to the normal distribution data for analysis, the nonparametric statistical method is used for comparison between groups. Nonparametric test was used to compare data between groups. Correlation analysis used Pearson correlation analysis. Covariance analysis was used to compare the differences in BPV between groups, and multiple stepwise regression analysis was performed on target organ factors related to BPV. *P* < .05 is statistically significant.

### 2.8 Ethics and dissemination

The study protocol was approved by the Fifth People's Hospital of Chengdu City. The approved protocol was registered. Each participant will be provided with information regarding the study protocol. Written informed consent will be obtained from each patient. The privacy of all participants will be protected. Personal medical records will be reviewed by investigators, who will promise to keep the content confidential. It will be performed in accordance with the standards of the International Committee on Harmonization on Good Clinical Practice and the revised version of the Declaration of Helsinki principles.

### 2.9 Protocol amendment

Any modifications to the protocol which have substantial effects on the conduct of the study, potential benefits to the patient, or impact on patient safety, including changes to eligibility criteria, patient population, sample size, study objectives, study design, and study procedures will require a formal amendment to the protocol. Such amendment will be sent to the Ethics Committee for further approval before implementation. Administrative changes of the protocol defined as minor corrections having no effect on the way the study is to be conducted will be documented and the Ethics Committee may also be notified of them.

## 3 Discussion

Hypertension is a major cause of many chronic and life-threatening diseases, such as cardiovascular disease and kidney failure. However, due to mild nonspecific symptoms, awareness is relatively low and patient compliance and control is poor. Hypertension is a complex syndrome affected by many genetic and environmental factors. Many epidemiological studies have shown a significant relationship between salt intake and blood pressure.^[12,13]^

BPV is associated with an increased risk of clinical TOD, such as cardiovascular and renal damage, and an increase in cardiovascular mortality.^[14]^

Blood pressure exhibits highly observed oscillations.^[15]^ There are 3 different types of BPV, depending on the time period in which it occurs: very short-term BPV, short-term BPV, and long-term BPV.^[16]^ Average consolidation rate (ARV) was introduced as a new BPV index. The ARV is the sum of the differences between every 2 consecutive readings. A systematic review of ARV assessment studies at 24 hour BPV was conducted in 19 studies, of which 17 reported a significant correlation between high ARV and TOD.^[17]^ In all these studies, BPV remained a significant independent predictor of all cardiovascular events.^[17]^ The increase of BPV in hypertensive patients is significantly correlated with the increase of TOD severity. Subsequent studies have shown that an increase in BPV is associated with an increased risk of cardiovascular events.^[18,19]^ It is speculated that TOD is not only associated with uncontrolled hypertension, but also with increased BPV. Whether the mechanism of TOD mediated by hypertension is caused by changes in blood pressure remains unclear. Our present protocol will provide a new sight in the association between among high salt intake, BPV, and target organ damage in patients with essential hypertension.

## Author contributions

Jianbo Lei orcid: 0000-0002-2073-1076.
